# Multi-omic analyses of hepatocellular carcinoma to determine immunological characteristics and key nodes in gene-expression network

**DOI:** 10.1042/BSR20211241

**Published:** 2021-07-12

**Authors:** Zhihui Wang, Shuijun Zhang

**Affiliations:** 1 Department of Hepatobiliary and Pancreatic Surgery, The First Affiliated Hospital of Zhengzhou University, Zhengzhou 450052, China; 2 Zhengzhou Key Laboratory of Hepatobiliary and Pancreatic Surgery and Digestive Organ Transplantation at Henan Universities, Zhengzhou 450052, China; 3 Open and Key Laboratory of Hepatobiliary and Pancreatic Surgery and Digestive Organ Transplantation at Henan Universities, Zhengzhou 450052, China; 4 Henan Key Laboratory of Digestive Organ Transplantation, Zhengzhou 450052, China

**Keywords:** gene expression, hepatocellular carcinoma, immune stratification, immune therapy, multi-omics

## Abstract

Hepatocellular carcinoma (HCC) is a common malignant tumor worldwide, but effective immunotherapy is still limited for those affected. Therefore, there is an urgent need to explore the specific mechanisms governing tumor immunity to improve the survival rate for those diagnosed with HCC. In the present study, we performed a new immune stratification of HCC samples into two subclasses (A and B) from The Cancer Genome Atlas and the International Cancer Genome Consortium databases, and comprehensive multi-omic analyses of major histocompatibility complex genes, gene copy-number variations, somatic mutations, DNA methylation, and non-coding RNAs. Subclass A was found to have a higher survival rate compared with subclass B, and there were significant immunological differences between the two clusters. Based on these differences, we identified DRD1 and MYCN as key hub genes in the immune-phenotype gene expression regulatory network. These results provide novel ideas and evidence for HCC regulatory mechanisms that may improve immunotherapy for this cancer.

## Introduction

The incidence of hepatocellular carcinoma (HCC) ranks sixth among malignant tumors, and it is the second leading cause of cancer-related deaths in the world, with more than 60,000 deaths per year [1,2]. For decades, significant progress has been made in the diagnosis and surgical treatment of HCC, and in radiotherapy and chemotherapy. However, due to the difficulty in diagnosing HCC early, its high recurrence rate, and associated distant metastasis, there have been no breakthroughs for reducing its mortality rate [3–6]. A number of emerging research areas have become the focus for trying to solve these cancer-related problems, such as regulation of methylation and glycosylation, and metabolism-related pathways [7–9]. However, the clinical prospects for these areas of research are not clear and are still far from any clinical applications.

At present, immunotherapy shows good prospects for HCC treatment. In recent years, researchers have found that CD8^+^ T-cell infiltrates are related to the prognosis of liver cancer [10,11]. Meanwhile, tumors infiltrated by immunosuppressive cells such as regulatory T cells (Tregs) tend to be associated with poor prognoses [12]. Recently, a major breakthrough in this field has been the development of the immune checkpoint inhibitor (ICI) anti-programmed cell death protein 1 (PD-1). This ICI can block the inhibition of an anti-tumor T-cell signaling pathway, thereby enhancing the body’s existing anti-tumor immune response. This method has proven its effectiveness in a variety of solid tumors, including those of HCC [13–16]. For example, the PD-L1 inhibitor atezolizumab has achieved unprecedented results in the treatment of patients with unresectable advanced HCC [17]. Up to now, six systemic therapies (atezolizumab plus bevacizumab, sorafenib, lenvatinib, regorafenib, cabozantinib and ramucirumab) have been approved for use by the FDA. Furthermore, the combined application of PD-L1 inhibitors and tyrosine kinase inhibitors is being explored. The breakthrough of ICIs heralds its great prospects in the field of HCC treatment [18,19]. However, only a small proportion of people respond to the anti-tumor effects of this ICI. A recent study has suggested that this may be due to the requirements for pre-existing T-cell infiltration and programmed death-ligand 1 (PD-L1) expression for the ICI to function [20]. In the above process, INF-γ played a key role regulating the expression of PD-L1, and in the identification of tumors with a cytotoxic immunophenotype [21,22]. In view of the complexity and dynamic nature of immune responses in the tumor microenvironment (TME), it remains difficult to predict a patient’s response to ICI therapy with certainty [23,24]. Okrah et al. used HCC RNA transcriptome data for immunotyping to predict HCC patient prognoses [25], but such efforts cannot predict the effectiveness of HCC immunotherapy, and the molecular mechanisms that establish and maintain immunophenotypes are still unknown. Therefore, there is an urgent need to better understand the HCC gene network regulating immune phenotypes and the associated molecular mechanisms. Here, we determined two classes of immunophenotype using unsupervised hierarchical analysis based on data from five immune system cells that were strongly correlated with PD-L1 and interferon gamma (IFNγ) expression. A comprehensive multi-omic approach using a variety of data (RNAseq, proteomics, and DNA methylation) was used to establish a gene regulatory network for HCC immune phenotyping, and to further establish key regulatory hub genes in the network. These regulatory hub nodes were strongly correlated with both the activation and suppression of immune responses. The present results can be used both for risk assessment and for predicting immunotherapy efficacy for HCC patients, and for the stratification of HCC patients according to possible immunotherapy benefits, all of which are significant for guiding the clinical management of HCC patients.

## Materials and methods

### Datasets for gene expression and clinical characteristics

All gene expression data and clinical follow-up information were obtained from The Cancer Genome Atlas (TCGA)-LIHC database (https://portal.gdc.cancer.gov/) and the International Cancer Genome Consortium database (ICGC-LIRI-JP) (https://dcc.icgc.org/). Protein expression data (based on TCGA-LIHC data) were obtained from The Cancer Proteome Atlas (https://www.tcpaportal.org/tcpa). The functional miRNA and lncRNA data (based on TCGA-LIHC data) were obtained from TCGA. These TCGA-LIHC datasets were used as the training cohorts, and ICGC-LIRI-JP datasets were used as the independent verification cohorts [26].

### CIBERSORT immune-cell scoring

The CIBERSORT tool assesses immune-cell infiltration by deconvoluting RNAseq expression matrix data from immune-cell subtypes based on the principle of linear support vector regression. We used this tool to calculate absolute immune-cell scores based on RNAseq expression data from the gene signatures of 22 immune cells (permutation = 1000).

#### Least absolute shrinkage selection operator (LASSO) and HCC analysis of subclass

We used the glmnet package in R software to reduce the dimensionality of TCGA-LIHC and ICGC-LIRI-JP datasets through a LASSO-Cox regression algorithm. We repeated the LASSO operation 100 times and removed immune-cell types that appeared more than 50 times among the 22 immune-cell types. The absolute values of the immune-cell scores were then converted using ln(*x* + 1). The aggregation was performed using Euclidean distance and Ward (unsquared distances).

### DNA methylation analysis

The DNA methylation dataset from TCGA-LIHC database was obtained as a download from https://gdc.xenahubs.net/download/TCGA-LIHC.methylation450.tsv.gz. After cleaning the data, we used the wateRmelon package in R software for standardization, and then assessed differential methylation using the minfi package in R software.

### TCGA-LIHC somatic mutation analyses

In the case of MutSig 2.0 q value < 0.05 and somatic mutation frequency > 5%, we compared the relative distribution of TCGA-LIHC candidate genes provided by cBioPortal (http://www.cbioportal.org/) among different clusters. The tumor map of somatic mutation pattern was performed by the R package “ComplexHeatmap”.

### Copy-number variation (CNV) analyses

Copy number variation (CNV) data was downloaded from http://www.firebrowse.org/. Subsequently, we used the CoNVaQ network tool to establish a statistical model of Fisher’s exact test (https://convaq.compbio.sdu.dk/). The CNV summary figure was generated by IGV_2.4.19, and the Circos diagram was drawn by the R software package ‘Rcircos’.

### Statistical analyses

Statistical analyses were performed using R software (> v. 3.5.1). For all comparisons, *P* values <0.05 were considered statistically significant.

## Results

### Immune subtypes of HCC samples based on PD-L1 and IFNγ expression levels

**Figure 1:**
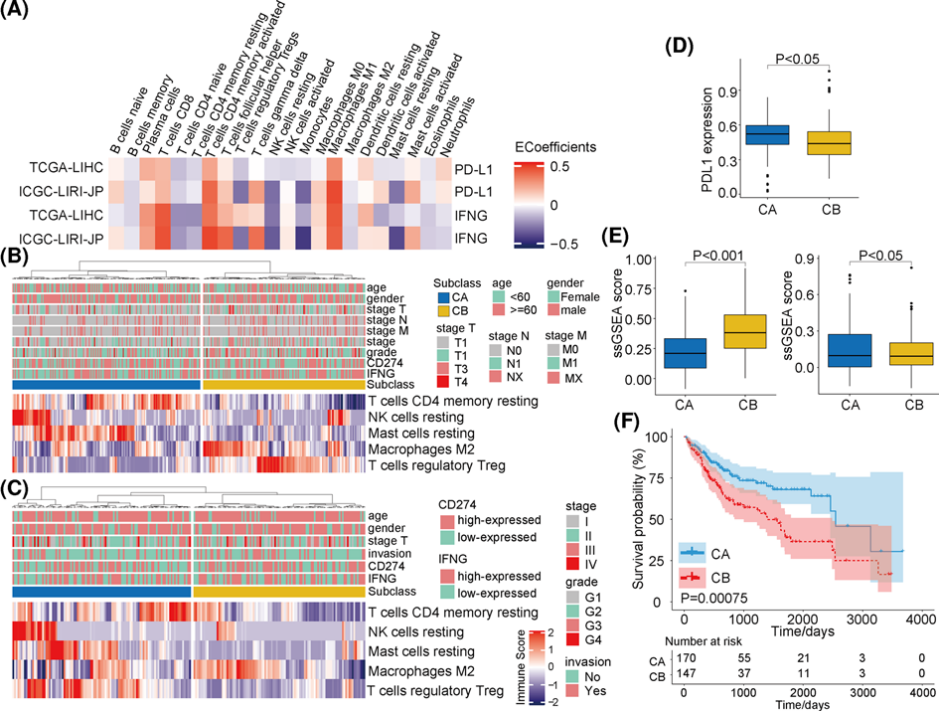
Immune subtypes of hepatocellular carcinoma based on PD-L1 and INFγ gene expression (**A**) Correlations between PD-L1, INFγ and immune-cell infiltration ratios in TCGA and the ICGC cohorts. (**B**) The distribution of immune subtypes and related clinical characteristics in TCGA cohort. (**C**) The distribution of immune subtypes and related clinical characteristics in the ICGC cohort. (**D**) Analysis of differences in PD-L1 protein levels between subclass A (CA) and subclass B (CB) in TCGA cohort. (**E**) Analysis of ssGSEA score differences in immune-related gene sets in TCGA and the ICGC cohorts between subclass A (CA) and subclass B (CB). (**F**) There was a significant difference in overall survival rate between subtypes.

There were 371 and 212 samples in the training cohort of TCGA-LIHC dataset and verification cohort of the ICGC-LIRI-JP dataset, respectively. Immune-cell scoring for each sample was determined using the CIBERSORT tool. We then used Spearman’s correlation method to calculate correlations between these immune-cell scores and PD-L1 expression levels. The results showed that 10 types of immune cells (resting memory CD4^+^ T cells, Tregs, resting mast cells, naive CD4^+^ T cells, monocytes, activated natural killer (NK) cells, M2 macrophages, memory B cells, and resting NK cells) were negatively correlated with PD-L1 expression levels (Figure 1A). The expression of INFγ, a PD-L1 transcription inducer secreted by activated T cells and NK cells, was positively correlated with M0 macrophages, follicular-helper T cells, M1 macrophages, CD8^+^ T cells, resting dendritic cells, activated memory CD4^+^ T cells, plasma cells, activated NK cells, and γδ T cells (Figure 1A). The subsequent LASSO-Cox regression calculations for these immune cells with strong PD-L1 and INFγ correlations determined that five immune-cell subtypes were significant: resting memory CD4^+^ T cells, Tregs, resting mast cells, resting NK cells, and M2 macrophages.

### Unsupervised hierarchical subclass analysis based on immune-cell subsets

Based on the above immune-cell subset obtained by the LASSO-Cox regression, we performed unsupervised hierarchical clustering on TCGA-LIHC cohort. Two resulting HCC sample clusters were identified: subclass A (CA) and subclass B (CB) (Figure 1B). Compared with CB, CA samples had higher levels of PD-L1 protein (Figure 1D). CB samples also showed more heterogeneity in the scoring of activated Tregs and M2 macrophages. Based on these results, CA was designated as an immunophenotype with high cytotoxicity and CB was designated as an immunophenotype with low cytotoxicity. Similar results were confirmed using the independent ICGC-LIRI-JP validation cohort (Figure 1C). In TCGA-LIHC and the ICGC datasets, we found that the single-sample gene set enrichment analysis (ssGSEA) scores for the extended immune gene signatures (EIGS) were significantly higher in CB compared with CA (Figure 1E), and there were no significant cluster-group differences for clinical characteristics. In addition, we found that the CA overall survival rate was significantly higher compared with CB (*P*=0.00075; Figure 1F).

**Figure 2:**
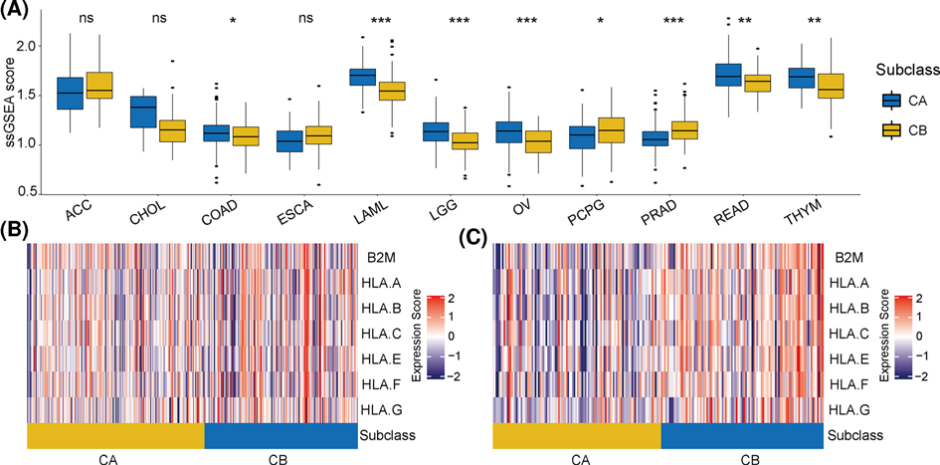
ssGSEA scores at the pan-cancer level, and the expression of MHC-related molecules between subtype A (CA) and subtype B (CB) (**A**) Differences in ssGSEA scores between CA and CB at the pan-cancer level. (**B**) Differences in the expression of MHC-related molecules between CA and CB in TCGA cohort. (**C**) Differences in the expression of MHC-related molecules between CA and CB in the ICGC cohort. *P<0.05, **P<0.01, and ***P<0.01

To verify the validity of these classification results, we also assessed these classifications at the pan-cancer level. After screening specific cohorts with low absolute immune-cell subpopulations scores, 11 cohorts were selected for analysis (ACC, CHOL, COAD, ESCA, LAML, LGG, OV, PCPG, PRAD, READ, and THYM). Unsupervised hierarchical clustering showed that classification of these tumor samples was similar to the results for TCGA-LIHC and the ICGC-LIRI-JP cohorts. For brevity, we have only included the clustering results for four of these tumor types (LAML, LGG, THYM, and ESCA) (Supplementary Figure S1). In addition, the ssGSEA enrichment scores of the CA clusters for these tumors were also higher than the scores of the CB clusters (Figure 2A).

### Immunophenotypes and major histocompatibility complex (MHC) class I gene expression differences

MHC class I molecules are involved in the recognition and presentation of foreign antigens in the body, the regulation of immune responses, and they play key roles in the process of presenting tumor antigens [27]. Here, we assessed any correlations between MHC class I molecules and immune clustering in TCGA-LIHC and the ICGC-LIRI-JP cohorts. The results showed that the expression levels of B2M and HLA were significantly higher in CA compared with CB (Figure 2B), indicating that CB tumor samples may escape immune surveillance through the decreased expression of MHC genes and may partly explain the poor prognoses of CB.

#### Differences in somatic mutations and CNVs between immune subtypes

Somatic mutation analyses of TCGA-LIHC cohort showed that CA and CB total mutation rates were not significantly different. Interestingly, the relative mutation frequencies of MUC16 in CA were significantly higher compared with CB, while that of TTN was lower than that of CB (Supplementary Figures S2 and 3). This result was also confirmed using the pan-cancer validation cohorts (ACC, CHOL, COAD, ESCA, LAML, LGG, OV, PCPG, PRAD, READ, and THYM) (Supplementary Figure S4).

**Figure 3:**
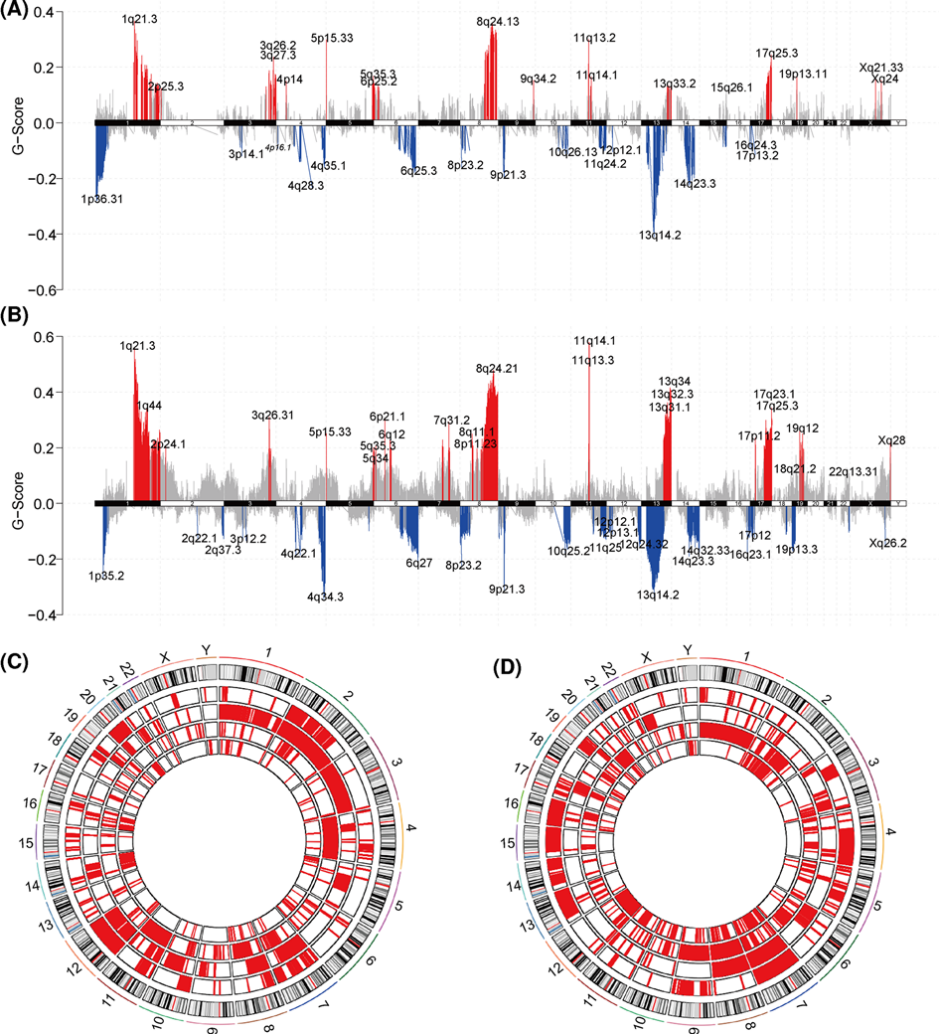
Differences in copy-number variations (CNVs) between subtypes (**A**) CNV statistics for subclass A (CA) in TCGA cohort. (**B**) CNV statistics for subclass B (CB) in TCGA cohort. (**C**) CNV statistics for CA in the pan-cancer cohort. From outside diameter to inside diameter: LIHC, OV, PRAD, and READ. (**D**) CNV statistics for CB in the pan-cancer cohort.

**Figure 4:**
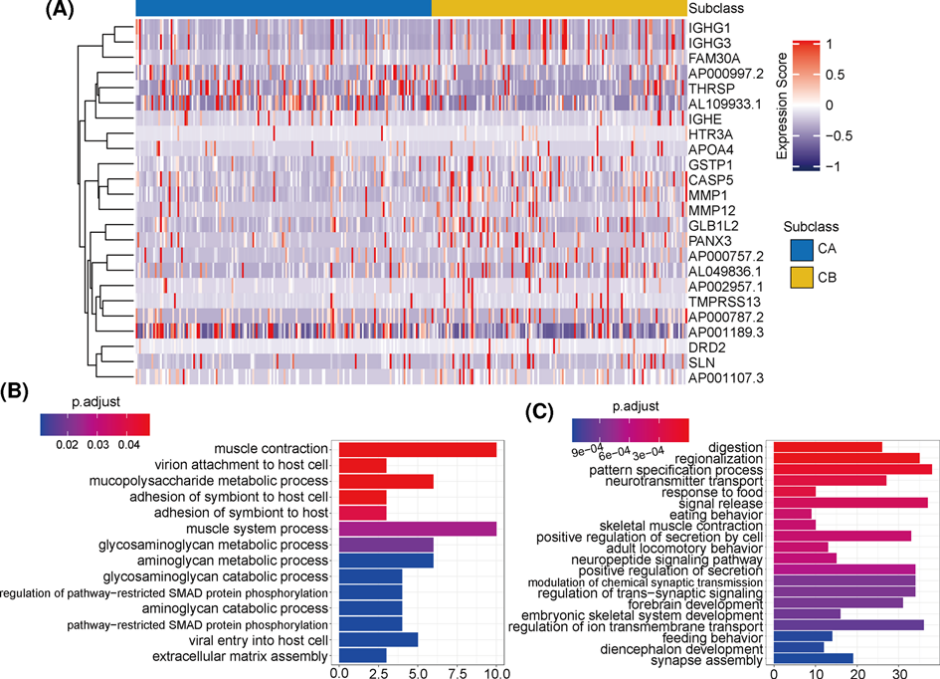
Analysis of methylation differences, and functional enrichment of pathways between subclasses (**A**) Analysis of differentially methylated gene expression between subclass A (CA) and subclass B (CB). (**B**) Functional pathway enrichment analysis of up-regulated genes in CA. (**C**) Functional pathway enrichment analysis of up-regulated genes in CB.

In addition, the analysis of somatic cell CNVs showed that in TCGA-LIHC cohort, there were increased gene copies (chromosomes 1q, 2p, 13q, and 17p) or genetic deletions (chromosomes 4q, 13q, 14q, and several hot spots in 19q) in CB compared with CA (Figure 3A,B). The CNV analyses of multiple solid tumor cohorts from TCGA (LIHC, OV, PRAD, and READ; from outside diameter to inside diameter) showed that the distributions of CNVs in the different cohorts were significantly different, involving different segments of multiple chromosomes (Figure 3C,D). These different mutation sites involved multiple chromosomes and are likely to extensively affect the transcription of many genes. In order to explore gene functionality, we screened the two cluster groups for differentially expressed genes (DEGs) and also performed a functional-enrichment analysis. The results showed that the up-regulated DEGs in CA were mainly concentrated among metabolic processes, such as aminopolysaccharide metabolism and mucopolysaccharide metabolism (Figure 4B), while up-regulated DEGs in CB were enriched in phylogeny and regulation of signaling pathways (Figure 4C). These results suggest that a possible reason for the poor prognoses of the low cytotoxicity cluster group (CB) was due to the inhibition of normal metabolism.

### DNA methylation differences between different clusters

Studies have shown that immune-activation genes can be epigenetically regulated through DNA methylation [28]. In order to explore the influence of DNA methylation on establishing and maintaining immune phenotype, we performed a genome-wide methylation analysis using TCGA-LIHC cohort. We screened a total of 33 regions showing differential methylation (FDR < 0.05), and a total of 25 gene promoter regions were identified as overlapping these differentially methylated regions (Figure 4A). CA regions of DNA with higher beta values than those in the same CB regions also showed gene expression levels that were relatively lower.

### Analysis of non-coding RNA differences between clusters

**Figure 5:**
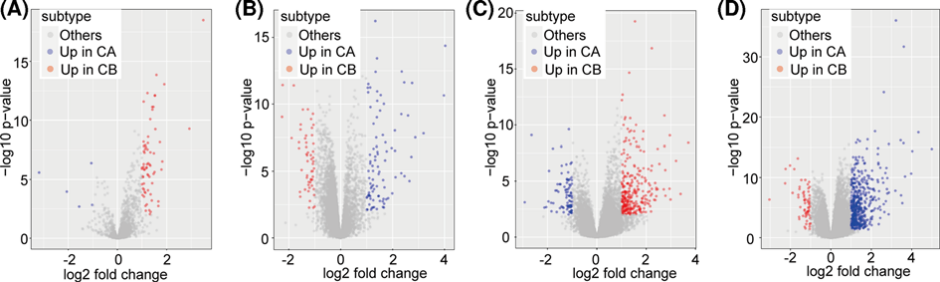
Volcano maps of differentially expressed miRNAs and lncRNAs (**A**) Volcano map of the miRNAs differentially expressed between subclass A (CA) and subclass B (CB). (**B**) Volcano map of differentially expressed lncRNAs between CA and CB. (**C**) Volcano map of lncRNA–miRNA interactions. (**D**) Distribution of differentially expressed genes and lncRNA–miRNA differential-expression links.

In the analysis of differentially expressed microRNAs in TCGA-LIHC cohort, we identified 74 miRNAs (fold change > 2, FDR < 0.05), and their predicted target-gene links totaled 64706. After filtering, we identified 148 links, of which 91 were up-regulated in CA and 57 were up-regulated in CB (Figure 5A). Similarly, we found 365 differentially expressed lncRNAs, of which 285 were up-regulated in CA and 80 were up-regulated in CB (fold change > 2, FDR < 0.05; Figure 5B). The miRNA target predictions produced 345 lncRNA-miRNA links, including 121 lncRNAs and 65 miRNAs (Figure 5C). Among the DEGs in the lncRNA–miRNA links, a 449 were up-regulated in CA, and 62 were up-regulated in CB (Figure 5D).

### Identification of key nodes in gene expression network

**Figure 6:**
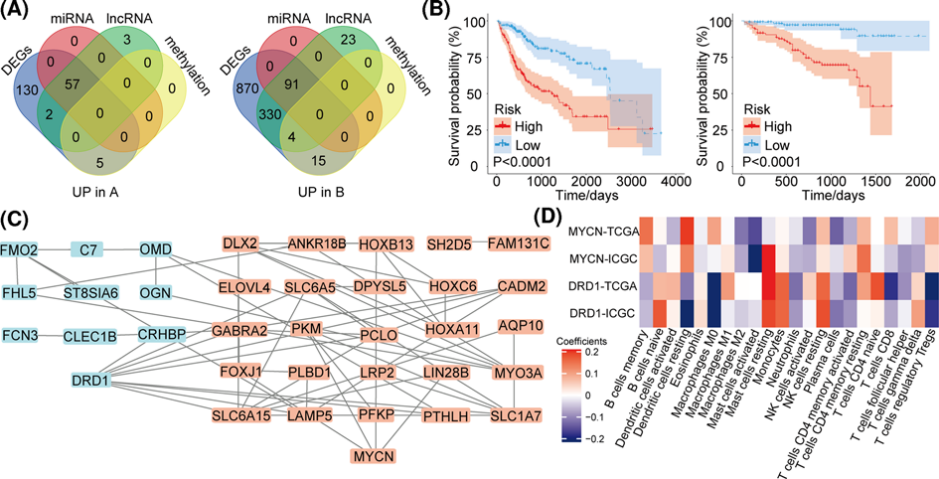
Identification of hub-gene nodes in the gene expression network (**A**) Statistical analysis of commonality between differentially expressed miRNAs, lncRNAs, differentially expressed genes, and differentially methylated genes for the two subclasses. (**B**) The risk-prediction abilities of the 44 prognosis-related genes. Left: TCGA cohort, right: ICGC cohort. (**C**) Protein–protein interaction (PPI) analysis emphasizes that MYCN and DRD1 were key nodes in the network. (**D**) Analysis of the correlation between MYCN and DRD1 in the training cohort and validation cohort and the type of immune cell infiltration.

In the above analysis, a total of 148 genes in TCGA-LIHC database were identified, of which 57 genes were up-regulated in CA and 91 genes were up-regulated in CB (Figure 6A). We performed a univariate Cox regression analysis using these genes to identify those significantly associated with HCC prognosis. A total of 44 genes were identified as relevant, of which 12 were up-regulated in CA and 32 were up-regulated in CB. The ssGSEA scores based on these 44 genes were determined using the training cohort and then confirmed in both the validation cohort and in other solid tumor cohorts. After using a multi-factor Cox regression method to calculate the risk scores for these 44 genes, we used the scores to analyze sample prognoses in the training dataset and the validation dataset, and found that these 44 genes had excellent prognostic value (*P*<0.0001, Figure 6B). We then used these same genes to perform a protein–protein interaction (PPI) analysis and found that MYCN and DRD1 were key hub-gene nodes in the PPI network (Figure 6C): DRD1 was highly expressed in CA and MYCN was highly expressed in CB. After analyzing any correlations between MYCN and DRD1 expressions and tumor immune-cell types, we found that the high expression of DRD1 in CA was positively correlated with immune activation, and that the high expression of MYCN in CB was positively correlated with immunosuppression. Lastly, we examined any correlations between immune-cell infiltration types and MYCN and DRD1 expression in both the training cohort and the validation cohort (Figure 6D).

## Discussion

The TME has been shown to both induce immune suppression and to promote tumor progression through multiple mechanisms [29]. The breakthrough of ICI treatment has garnered well-deserved attention, but as mentioned above, only a small percentage of patients respond to it [30,31]. Revealing the immune-phenotype characteristics of the TME and the complex mechanisms for its establishment and maintenance are key issues that must solved to better understand cancer treatments. Here, differences in immune-cell infiltrations among HCC tumor samples provided for a new tumor-sample stratification, and relevant results using it were verified using other tumor cohorts. Furthermore, we used a multi-omics approach to perform an overall analysis that included epigenetics and mutations between the two clusters. Lastly, we identified DRD1 and MYCN as key hub genes in the HCC regulatory network based on immune phenotyping.

The CIBERSORT tool was used to evaluate immune-cell scores from hundreds of HCC samples using TCGA-LIHC and the ICGC cohorts. Based on these scores, we screened immune-cell types that were highly correlated with the expressions of PD-LI and INFγ. The LASSO-Cox regression analysis further determined prognosis-related candidate immune-cell types: T-cell subtypes, CD4 subtypes, Treg subtypes, mast-cell subtypes, NK-cell subtypes, and macrophage subtypes. Using an unsupervised hierarchical subclass analysis, we identified two main clusters: a highly cytotoxic immunophenotype (CA) with higher expression levels of PD-L1 and INFγ transcripts, and PD-L1 protein. This CA group of HCC patient samples also had significantly better prognoses compared with the CB group of patient samples. These results were also verified in 11 other patient tumor-sample cohorts. Overall, these results demonstrate that our hierarchical clustering model was effective and consistent.

MHC class I molecules are key for tumor-antigen presentation and crucial for tumor immune surveillance [32,33]. A decrease in MHC class I expression may mean that tumors are more likely to escape immune surveillance, thereby promoting tumor progression. Interestingly, in our analysis, the CA expression levels of B2M and HLA were significantly higher than those in CB for both the training and the validation cohorts of HCC samples. This immunophenotype difference may partially explain the different prognoses for the two clusters.

In addition, the decreased somatic mutation rate of MUC16 and the increased rate of TTN somatic mutations were significant features of CB. A recent study has shown that MUC16 mutations may be related to the efficacy of ICI therapy and to the improvement of prognosis-related genomic factors in solid tumors [34]. A possible mechanism for this is that MUC16 binds to the Siglec 9 receptor on NK cells, downregulating their cytotoxicity and thereby allowing tumor cells to escape immune surveillance [35]. In addition, studies have confirmed that TTN mutations are enriched in samples with high immunostimulatory characteristics [36]. The TTN-AS1 axis has also been reported to be tumor-promoting in a variety of cancers including prostate cancer, HCC, and breast cancer [37–39], and a decrease in its activity can inhibit tumor aggressiveness. Davoli et al. reported that CNVs may be related to immune-evasion markers and a decreased response to immunotherapy [40]. Our analysis of somatic CNVs showed that CB had increased copies of chromosomes 1q, 2p, 13q, and 17p, and a loss of copies for chromosomes 4q, 13q, 14q, and 19q. These transcription-affected genes are involved a wide range of functions, and the DEGs up-regulated in CA were mainly enriched in metabolic pathways. While interactions between metabolic processes and the TME are well known, and changes in the immune status of the TME can affect tumor metabolism and lead to changes in tumor biological behavior [41–43], clear mechanisms for this have not been demonstrated. The present results suggest that poor CB prognoses may be due to the inhibition of normal metabolism caused by differences in immune status [43], but this hypothesis requires further research to validate.

By combining the genes identified in the above analyses with a Cox prognostic analysis, we identified 44 genes with significant prognostic values. Their predictive effectiveness for prognoses was verified using the risk scores calculated using a multivariate Cox regression. We further identified both MYCN and DRD1 as key hub-gene nodes in the HCC PPI network, with DRD1 being highly expressed in CA and MYCN being highly expressed in CB. Their expression levels are likely to regulate the expression of other genes in this HCC network, but as a dopamine receptor gene, there is no clear evidence that DRD1 is related to cancer. Interestingly however, Ostadali et al. found that DRD1 expression was strongly correlated with the immune system, and that lymphocytes expressed DRD1 at high levels [44]. However, conclusive evidence that these receptors play a role in lymphocytes remains unclear. The immunosuppressive effect of MYCN expression in cancers, including neuroblastoma and lung cancer, is well known [45–47], and Masso-Valles et al. reported that MYCN inhibition may be a potential cancer immunotherapy [45]. Consistent with this, our CB sample group showed high expression levels of MYCN, and this expression was associated with immunosuppression that may be an important factor for tumors escaping immune surveillance. This also emphasizes, once again, that MYCN inhibitors may be a significant opportunity to enhance the effects of immunotherapy.

Exploring the heterogeneous response of ICIs at the genetic level helps to accurately identify sensitive HCC patients to reduce unnecessary costs and avoid wasting precious time. Due to the complexity of the immune response in the tumor microenvironment, although a lot of work has been done to try to illustrate the process, the current progress is still not satisfactory. Our research attempts to provide a new perspective to explain the process and preliminarily prove the importance of MYCN and DRD1 in the immune response. However, it is a systematic project to fully understand the heterogeneous response of HCC patients to ICIs [19]. Therefore, as mentioned above, at the current stage, the combined application of ICIs, tyrosine kinase inhibitors (TKIs) and anti-angiogenic drugs is a very valuable study for improving the prognosis of HCC patients.

In general, our study explored the differential immune response of HCC patients based on transcriptome data from a large sample set. However, the present study has some limitations. The mechanism of the immune response of DRD1 and MYCN needs to be confirmed by molecular experiments in HCC. The possibility of its clinical application still needs to be further explored.

## Conclusion

In the present study, we identified a new and effective immune stratification for HCC through multi-omics data analyses and demonstrated immunological differences between HCC sample clusters. Based on these differences, we determined that DRD1 and MYCN are the key hub genes nodes of the immune-phenotypic gene expression regulatory network, increasing our understanding of possible HCC mechanisms for improving immunotherapy.

## Supplementary Material

Supplementary Figures S1-S4

## Data Availability

The data that support the findings of this study are available from the corresponding author upon reasonable request.

## Impact Statement

In this study, we identified a new and effective immune stratification for HCC through multi-omics data analyses and demonstrated immunological differences between HCC sample clusters. Based on these differences, we determined that DRD1 and MYCN are the key hub genes nodes of the immune-phenotypic gene expression regulatory network, increasing our understanding of possible HCC mechanisms for improving immunotherapy.

## Abbreviations

DEG: differentially expressed gene
HCC: hepatocellular carcinoma
ICI: immune checkpoint inhibitor
MHC: major histocompatibility complex
PD-1: programmed cell death protein 1
PD-L1: programmed death-ligand 1
TME: tumor microenvironment
Treg: regulatory T cell
