# Supplementary material for: Multi-omic analyses of hepatocellular carcinoma to determine immunological characteristics and key nodes in gene-expression network
Source: Biosci Rep. 2021 Jul 12;41(7):BSR20211241. doi: 10.1042/BSR20211241 (PMC8276092; doi:10.1042/BSR20211241)
Supplement: Supplementary Figures S1-S4 [file BSR-2021-1241_supp.pdf]

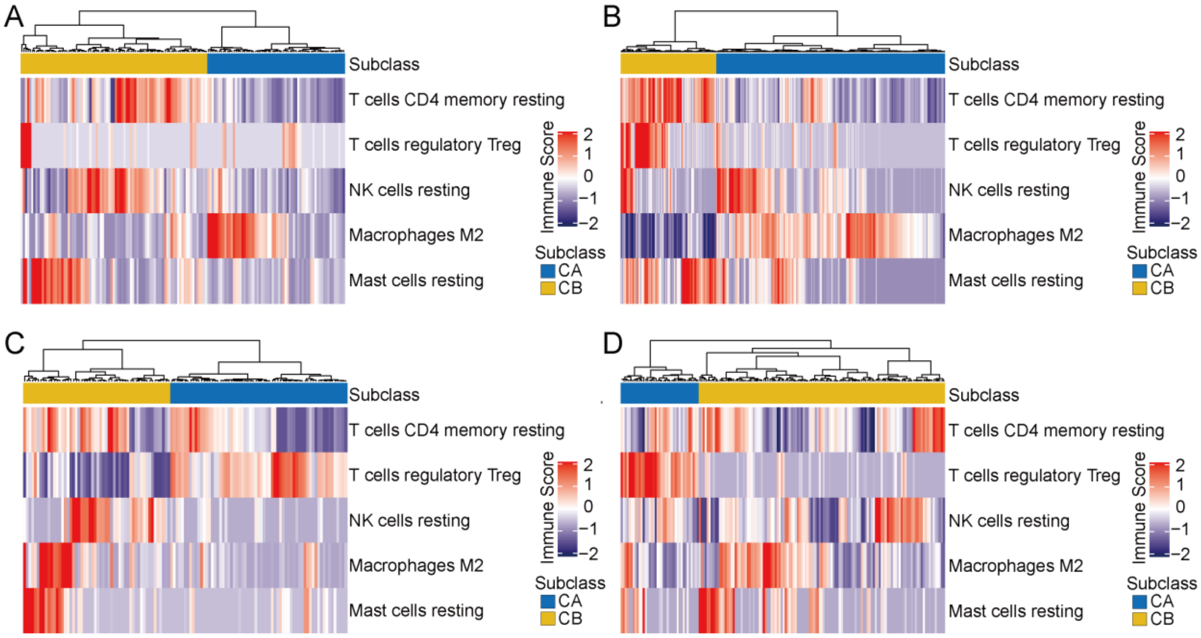

Supplementary Figure 1. Verification of subtype classification at the pan-cancer level.

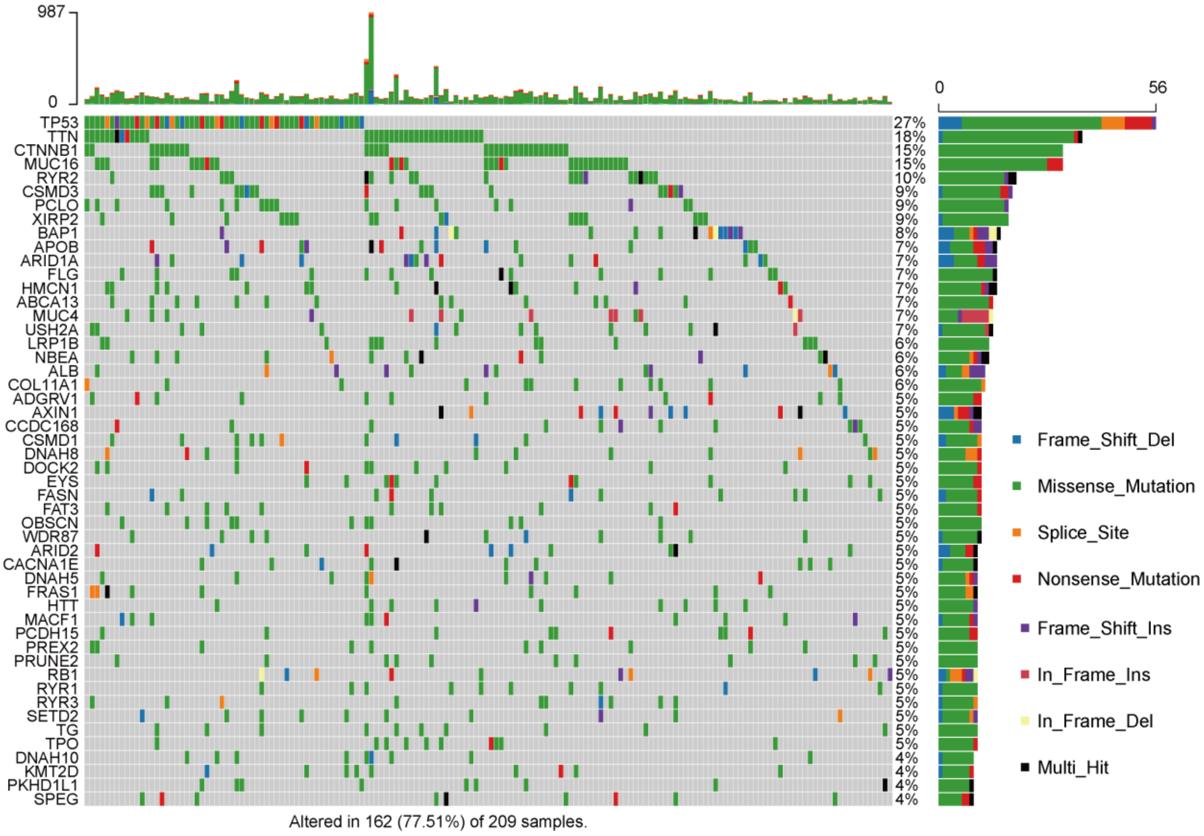

Supplementary Figure 2. Somatic mutation analysis of TCGA-LIHC cohort.

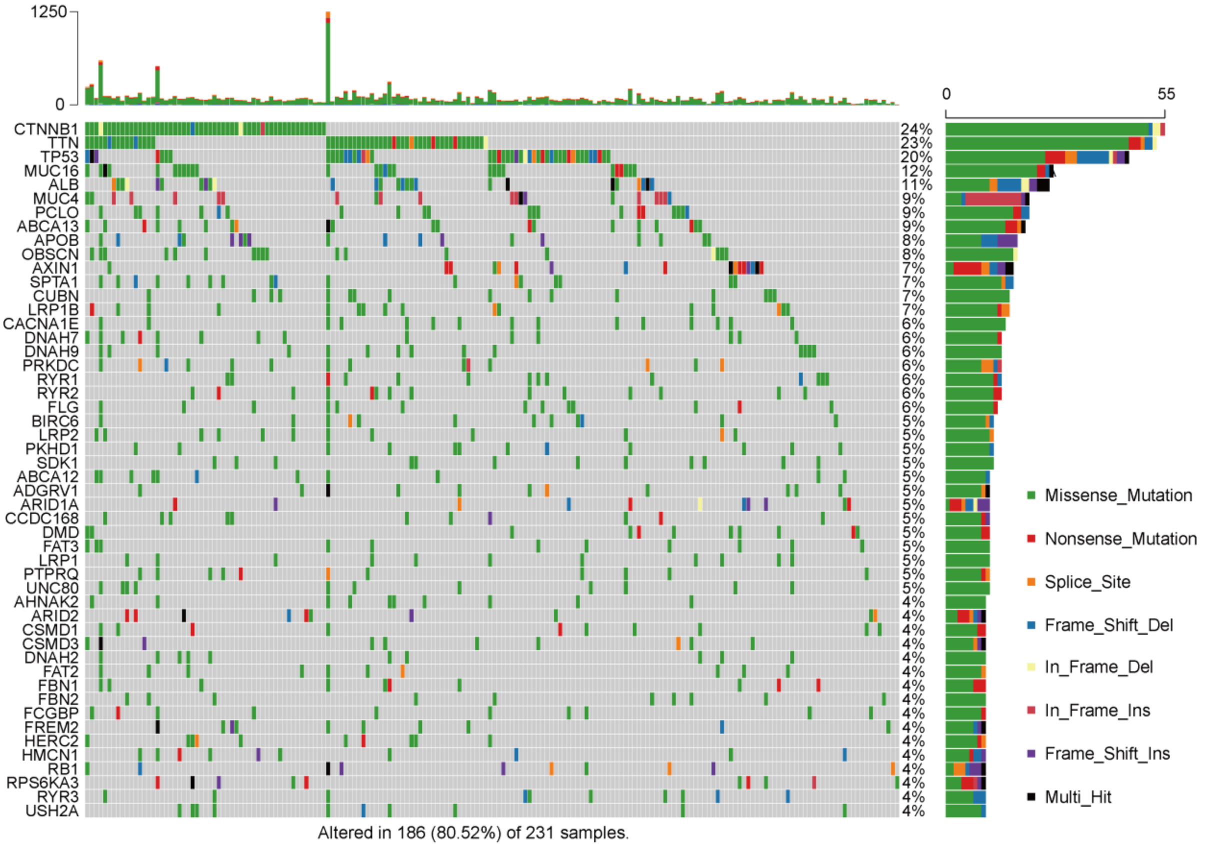

Supplementary Figure 3. Somatic mutation analysis of the ICGC cohort.

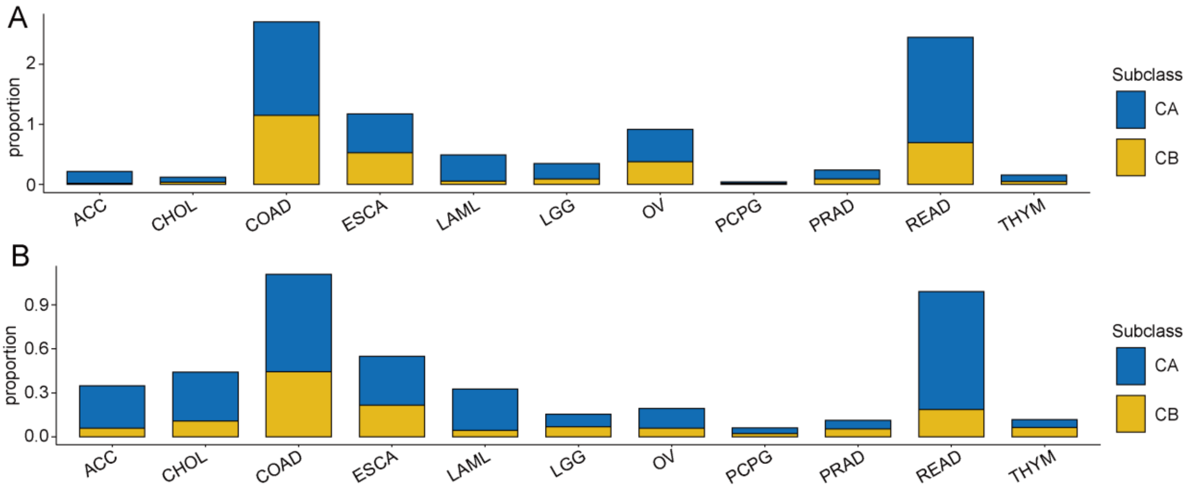

Supplementary Figure 4. Distribution statistics for the number of MUC16 and TNM gene mutations across the pan-cancer. (A) Distribution statistics for the number of MUC16 gene mutations in subclasses A and B across the pan-cancer level. (B) Distribution statistics for the number of TNN gene mutations in subclasses A and B across the pan-cancer level.
